# Attitudes and perceptions of next-of-kin/loved ones toward end-of-life HIV cure-related research: A qualitative focus group study in Southern California

**DOI:** 10.1371/journal.pone.0250882

**Published:** 2021-05-07

**Authors:** Sogol S. Javadi, Kushagra Mathur, Susanna Concha-Garcia, Hursch Patel, Kelly E. Perry, Megan Lo, Jeff Taylor, Andy Kaytes, Susan Little, Sara Gianella, Davey Smith, Karine Dubé

**Affiliations:** 1 AntiViral Research Center, University of California San Diego, San Diego, CA, United States of America; 2 HIV Neurobehavioral Research Center, University of California San Diego, San Diego, CA, United States of America; 3 UNC Gillings School of Global Public Health, Chapel Hill, NC, United States of America; 4 AntiViral Research Center Community Advisory Board, San Diego, CA, United States of America; 5 HIV + Aging Research Project–Palm Springs (HARP + PS), Palm Springs, CA, United States of America; 6 Division of Infectious Diseases and Global Public Health, University of California San Diego, La Jolla, CA, United States of America; Newcastle University, UNITED KINGDOM

## Abstract

As end-of-life (EOL) HIV cure-related research expands, understanding perspectives of participants’ next-of-kin (NOK) is critical to maintaining ethical study conduct. We conducted two small focus groups and two one-on-one interviews using focus group guides with the NOK of Last Gift study participants at the University of California, San Diego (UCSD). Participating NOK included six individuals (*n* = 5 male and *n* = 1 female), including a grandmother, grandfather, partner, spouse, and two close friends. Researchers double-coded the transcripts manually for overarching themes and sub-themes using an inductive approach. We identified six key themes: 1) NOK had an accurate, positive understanding of the Last Gift clinical study; 2) NOK felt the study was conducted ethically; 3) Perceived benefits for NOK included support navigating the dying/grieving process and personal growth; 4) Perceived drawbacks included increased sadness, emotional stress, conflicted wishes between NOK and study participants, and concerns around potential invasiveness of study procedures at the EOL; 5) NOK expressed pride in loved ones’ altruism; and 6) NOK provided suggestions to improve the Last Gift study, including better communication between staff and themselves. These findings provide a framework for ethical implementation of future EOL HIV cure-related research involving NOK.

## Introduction

In the United States today, the majority of people living with HIV (PLWHIV) are no longer dying of AIDS-defining illnesses. Instead, they are living longer and dying of chronic conditions similar to those of the general population, such as non-AIDS-defining cancers, cardiovascular disease, and neurodegenerative diseases [1]. The Last Gift is an end-of-life (EOL) HIV cure-related research study at the University of California, San Diego (UCSD) [2,3]. The study is part of the National Institute of Allergy and Infectious Diseases (NIAID)’s and the National Institute of Mental Health (NIMH)’s initiative to increase research involving terminally ill PLWHIV. As a rapid autopsy research study, the Last Gift enrolls altruistic PLWHIV who are terminally ill secondary to a non-AIDS-defining illness. Participants have a prognosis of less than 6 months. In the ante-mortem phase of the study, participants undergo routine blood draws and optional tissue collection. This phase also includes in-depth socio-behavioral interviews with both participants and their next-of-kin/loved ones/intimate partners (hereafter referred to as ‘NOK’) regarding their experiences and feelings toward EOL HIV cure research. In the post-mortem phase, a rapid autopsy is performed within six hours of death. Participants who enroll in the study understand that the study will not confer direct clinical benefits and will not cure them of their HIV or their terminal illness.

Antiretroviral therapy has reduced and prevented HIV mortality but cannot eliminate HIV from the body since the virus hides in latently infected cells [4]. Furthermore, studying animal models is insufficient for finding a cure for HIV because human testing remains necessary to prove safety and efficacy of interventions in humans [4]. As such, to better understand HIV persistence and potentially eliminate hidden HIV reservoirs from the human body, Last Gift researchers proposed “a perimortem translational research model” to identify where HIV persists inside the body [4]. Therefore, the rationale for enrolling terminally ill PLWHIV in HIV cure-related research is six fold: "1) the absence of any reasonable expectation of direct clinical benefits in most HIV cure-related research, 2) the manifest desire in this community to ‘give back’ to the HIV research field, 3) limited opportunities for terminally ill PLWHIV to participate in HIV clinical research in general, 4) the fact that people at the EOL may be willing to accept higher risks for research participation, 5) the possibility for donating their full body for a rapid research autopsy, and 6) the opportunity to create a new translational research model to advance HIV cure science (e.g. to test novel HIV cure-related interventions in a human model) [5].”

Using blood and tissue samples collected before and after death from six Last Gift study participants, Chaillon and colleagues found that HIV persists in blood cells and across 28 deep tissues including cardiovascular, gastrointestinal, and lymphoid tissues [6]. Rawlings et al. developed a rapid research autopsy protocol to guide the collection of fluids and tissues for interrogating HIV reservoirs [7].

NOK are an integral part of the Last Gift study. Within studies in EOL cancer research, respect and cooperation with NOK have been described as a key ethical consideration [8,9]. Similarly, understanding and respecting the attitudes and experiences of participants’ communities (including NOK) in EOL HIV cure-related research is critical for ethical study conduct [5,10]. In studying NOK perspectives, we seek to safeguard vulnerability, ensure acceptability from all stakeholders, and center the study around participants and their NOK. We conducted focus groups to better understand NOK’s perspectives of the Last Gift study and its impact on them and participants. The focus groups also sought to elucidate ethical concerns involving NOK. In this paper, we report results from small focus groups conducted with NOK in 2019, two years following the initiation of the Last Gift study in 2017.

## Methods

NOK were identified by Last Gift study participants (LG) who referred them into our qualitative study. NOK provided both written and verbal informed consent for their own research involvement. A total of six NOK participated in the focus group component of the Last Gift study. Two NOK participated in each of small focus groups #1 and #2. Two remaining NOK preferred answering questions from the focus group guide via one-on-one interviews. Focus group recruitment was conducted via emails and phone calls from February 2019 through July 2019. Focus groups lasted between 60–90 minutes. One focus group and two interviews took place at the AntiViral Research Center (AVRC); however, two participants were unable to find transportation, therefore, the second focus group took place at their home. Only the participants and research staff were present during the discussions. Participants were reimbursed $50 USD for their participation.

A study staff member (K.D.) developed the focus group guide in collaboration with the AVRC Community Advisory Board and the Stakeholder Advisory Board of HIV + Aging Research Project-Palm Springs. We presented drafts of focus group guides to AVRC Community Advisory Board members and revised them using an iterative process according to their suggestions. The Last Gift Study and focus group guides were approved by the UCSD Institutional Review Board (IRB).

The small focus groups setting created an opportunity for NOK to share collective experiences in a safe environment, removing the sense of isolation following the death of a loved one, and providing richness in information gathered [11]. Smaller focus groups encourage greater depth in data collection [12]. Additionally, by limiting focus group size, discussions more accurately mirrored ordinary conversation, much like dyadic interviews [12]. Drawbacks of focus groups include the possibility of groupthink, bias in the researchers’ data interpretation, social desirability, and reluctance to share negative experiences in group settings [11].

Focus group questions covered perceptions and concerns about the Last Gift study, coping strategies to mitigate stress and grief associated with the EOL process and the study, ethical considerations for the study, and recommendations for study improvement (**Table 1)**.

**Table 1:** Small focus group question route with next-of-kin/loved ones of last gift study participants (Southern California, 2019).

**Introductory/General Questions** • First, thank you so much for your time. • What does the Last Gift study mean to you? • What do you think the Last Gift study means/meant to the study participants? **Focus Group Discussion Questions for Next-of-Kin/Loved Ones of Last Gift Participants** For All Next-Of-Kin/Loved Ones • What does it mean to you to be part of the study as a next-of-kin/loved one? • What are your feelings about the Last Gift study? • Has the Last Gift study changed your life? If yes, how so? • How do you/did you manage the stress associated with the end-of-life process? • Do/did you see any benefits/positives to the Last Gift study participant of being in the study? • Do/did you see any risks/negatives to the Last Gift study participant of being in the study? For Next-of-Kin/Loved Ones of Last Gift Participants Who Are Still Alive • How did you react to the Last Gift participant’s decision to enroll in the study? • Can you discuss some of the issues that you are going through at the moment? • What are you doing to cope with the end-of-life process of the Last Gift study participant? • Do you have any concern(s) about the Last Gift study? If so, can you please tell us what they are? For Next-of-Kin/Loved Ones of Last Gift Participants Who Have Passed Away • Did you experience grief as a result of the Last Gift participant’s passing? • Has the Last Gift study helped with the bereavement or grieving process? If so, in what way(s)? • What did you do to cope with the loss of the Last Gift study participant? • How was your experience with the Last Gift body donation process? Did you have any difficulty letting go of the body of the Last Gift study participant? • Did you have any concerns about the Last Gift study? If yes, what are they? • Did you experience any negative effect (i.e. stigma) as a result of losing a loved one to HIV? If yes, can you please explain? • Did you commemorate the life of the Last Gift study participant in any way? If yes, in what way(s)? • What would you say to future next-of-kin/loved ones of Last Gift study participants? Ethical Considerations • Do you see any ethical issue with the Last Gift study? • We often worry that people who are terminally ill are a vulnerable population. Do you consider the Last Gift study participants to be a vulnerable group? • Do you feel that the Last Gift study team acknowledged the loss you are experiencing/have experienced? **Ending Questions** • Do you have any recommendation to improve the conduct of the study? • Can you think of anything else you would like to share with the group on this topic?

Two participants’ (NOK of Last Gift participant [LG]04 and LG08) involvement in focus groups was not possible. One participant was out of the country and the other was grieving the recent death of his loved one. Thus, out of respect to NOK, the study team remained flexible and conducted one-on-one interviews using focus group guides, as NOK had previously provided written informed consent to share their perspectives as part of a small focus group. Repeat interviews were not necessary for our study.

The members of our research team hold M.D., DrPH, MPH, and B.S. and B.A degrees. Both male- and female-identifying researchers contributed to this study. Research staff members (S.S.J. and K.M.) took detailed notes during the focus groups (and two interviews). Focus group facilitators (K.D. and S.S.J.) with prior experience in moderating focus groups ensured that participants had equal opportunities to express their thoughts. The focus groups (and two interviews) were audio-recorded and a member of the study team (S.S.J. or K.D.) uploaded audio files into a secure server. Study team members (S.S.J., K.M., and H.P.) transcribed each interview verbatim using Microsoft Word and removed identifiers. Research team members (S.S.J and H.P.) reviewed transcripts for completeness and quality assurance. We did not return transcripts to NOK for verification to avoid further distress to NOK. Study staff then deleted source audio files from the secure server in accordance with the IRB-approved informed consent form and work instructions. Socio-behavioral researchers and focus group facilitators were separate from the Last Gift study’s clinical research team and therefore did not know the participants prior to the sessions.

We followed a multidisciplinary theoretical framework integrating biomedical, socio-behavioral, ethics, and community-engaged domains that are recommended for HIV cure-related research [13,14]. Researchers (S.S.J. and K.M.) double-coded the transcripts manually for overarching themes and sub-themes using an inductive approach [15,16]. First-order codes included analyzing NOK statements by distinguishing repetitive phrases and/or ideas, identifying examples, and analyzing transitions in themes within the questions themselves [16]. We also used a phenomenological approach to study NOK narratives and understand their lived experiences without a predetermined framework [17], as very little was known about how NOK experience EOL HIV cure-related research participation. Given the novelty of EOL HIV cure-related research, our study did not use a pre-existing coding scheme. Key themes and relevant quotes were organized into a structured format in Microsoft Word. Salient quotes have been included in the Results section with supplementary quotes included in **S1 Appendix**. This study is reported according to Consolidating Criteria for Reporting Qualitative Research (COREQ) guidelines. Our checklist included in the **S2 Appendix**.

## Results

This paper provides insight from six NOK (*n* = 5 male and *n* = 1 female) who consented to participate in the small focus group component of the Last Gift study. These NOK are representative of five Last Gift study participants (LG01, LG04, LG05, LG07, and LG08). Two NOK for LG07 agreed to participate in the focus group. The NOK of LG04 and LG08 participated in one-on-one interviews using focus group guides, as they were unable to attend scheduled focus group sessions. Two LG participants, LG02 and LG06, chose to forego referring a NOK to the study. Participating NOK included a grandmother, grandfather, partner, spouse, and two close friends (**Table 2**). The focus groups (and two interviews) were conducted between May and July 2019. Of the NOK involved with the Last Gift Study, LG03’s NOK, LG04’s second NOK, and LG05’s second NOK declined to participate in the small focus group discussions (**Table 3**).

**Table 2:** Demographic characteristics of NOK/loved ones who responded to focus group guide questions (San Diego, California, 2019).

Participant Number	Sex	Race/Ethnicity	Relationship to Last Gift Study Participant	Focus Group Number
LG-01-NOK	Male	White	Spouse	Focus Group #1
LG-04-NOK	Male	White	Close Friend	One-on-One Interview (Using Focus Group Guides) #1
LG-05-NOK	Male	Hispanic/Hispanic Descent	Partner	Focus Group #1
LG-07-NOK-I	Female	White	Grandmother	Focus Group #2
LG-07-NOK-II	Male	White	Grandfather	Focus Group #2
LG-08-NOK	Male	White	Close Friend	One-on-One Interview (Using Focus Group Guides) #2

**Table 3:** Reasons NOK/loved ones did not participate in focus groups or one-on-one interviews (using the focus group guide).

Participant Number	Sex	Race/Ethnicity	Relationship to Last Gift Participant	Reason for Not Participating
LG-02-NOK	N/A	N/A	N/A	LG02 did not refer any NOK and relayed that he did not want to burden his parents who lived in a different state
LG-03-NOK	Male	White	Spouse	Unavailable at the time of the focus groups and was too bereft to participate in a one-on-one interview
LG-04-NOK-II	Male	White	Close Friend	Out-of-state at the time of the focus groups and was too bereft to participate in a one-on-one interview
LG-05-NOK-II	Female	White	Sibling	Did not want to participate in focus groups
LG-06-NOK	N/A	N/A	N/A	LG06 did not refer any NOK and relayed that he had no connections with his out-of-state NOK

**Fig 1:**
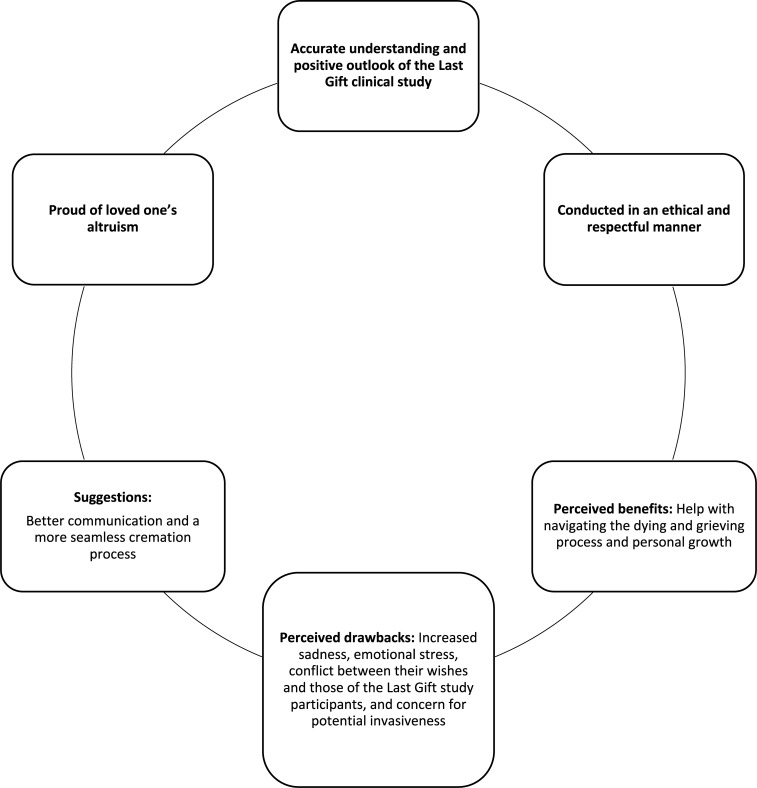
Summary of the six emerging themes from discussions with NOK.

**Fig 2:**
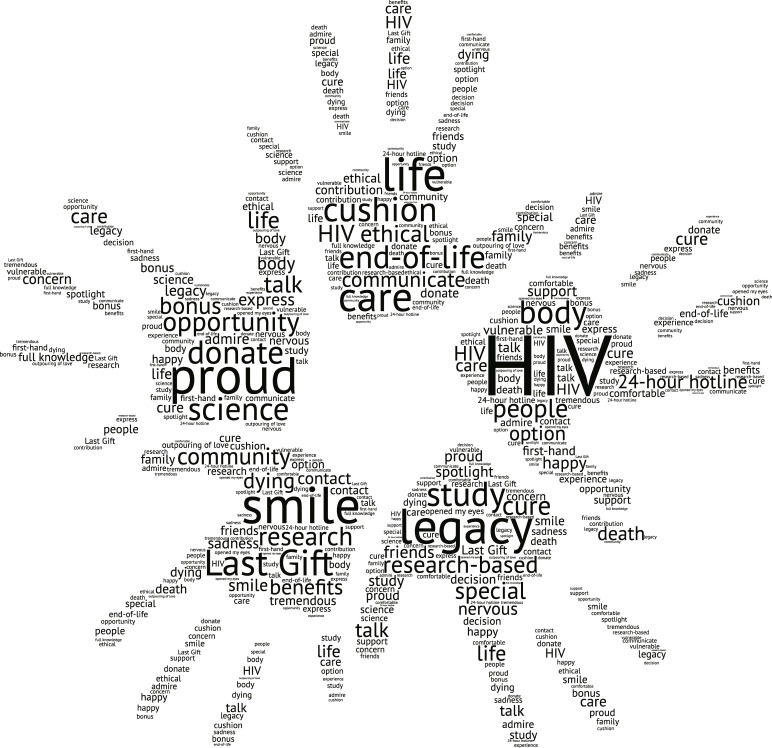
Word clouds from NOK quotes from discussions with NOK on HIV cure-related research at the EOL (San Diego, California, 2019).

Our thematic analysis extracted six themes from the focus groups (and two interviews): 1) NOK had an accurate and overall positive understanding of the Last Gift clinical study; 2) NOK felt the Last Gift study was conducted in an ethical and respectful manner; 3) Support with navigating the dying and grieving process and personal growth were noted by NOK to be perceived benefits associated with their participation in the Last Gift study; 4) Perceived drawbacks for NOK included increased sadness and emotional stress, conflicted wishes between NOK and study participants, and concerns around potential invasiveness of study procedures at the EOL; 5) NOK expressed pride in their loved ones’ altruism for participating in the Last Gift study and creating further meaning in their EOL process; and 6) NOK identified suggestions to improve Last Gift study implementation, such as better communication between staff and NOK as well as a more seamless cremation process following rapid research autopsy. **Fig 1** graphically illustrates these emerging themes. To capture the nuances of our discussions, we summarized common words used by participating NOK in **Fig 2**.

### Meaning of the last gift study

There was an accurate understanding among NOK of the Last Gift study’s goal of identifying hidden viral reservoirs that lead to HIV persistence. NOK recognized that Last Gift study participants were not undergoing therapeutic and/or curative procedures for HIV or their terminal illness.

“*[The study] provides an opportunity for people that are at end-of-life to donate to science their body and to help further the research and study of HIV and its effects on how it affects people*.”–LG-01-NOK (Spouse) Focus Group #1.

“*[The Last Gift] is a research-based study that looks at how HIV is stored in your body long term and then at the end of someone’s life*, *after they sign up*, *their tissues are donated*. *And the results are added to a larger study of what happens to people that are living with years of the HIV*.”–LG-08-NOK (Close Friend) One-on-One Interview #2.

When asked about the Last Gift team’s role, some NOK noted that they saw the Last Gift clinical staff as being part of their care team.

“*Once [LG01] was diagnosed with ALS [amyotrophic lateral sclerosis]*, *we never saw his primary care*, *so having the Last Gift group take care of him was special to him*.”–LG-01-NOK (Spouse) Focus Group #1.

NOK viewed the Last Gift study as a positive opportunity offered to PLWHIV at the EOL. In fact, some NOK stated that they, too, would participate in the study if given the opportunity.

“*I think this is something I would do for myself if the study is still going*.”–LG-04-NOK (Close Friend) One-on-One Interview #1.

Overall, NOK had an accurate understanding of the Last Gift clinical study. NOK were aware that the study would not confer any direct benefits or cures to their loved ones’ HIV and/or terminal illness. One NOK also stated their loved one appreciated the support they received from the study’s clinical staff, but also perceived them to be part of their clinical care team. NOK perceived the study in a positive light and saw it as an opportunity that they would potentially want to enroll in themselves.

### Ethical conduct of the last gift study

NOK did not perceive any ethical issues associated with the Last Gift study. They also felt the study was conducted ethically and respectfully.

“*No [ethical issues]*. *I think the way you guys handle everything is really well and you’re very open with the whole process and the participants have the choice at any time to opt-out so I see*, *I saw no ethical issues*.”–LG-05-NOK (Partner) Focus Group #1.

*“I don’t think*, *he didn’t feel that his arm was being twisted or [the study] was something that he didn’t want*. *And certainly*, *the added benefits of the support in dying was such an unexpected bonus for him and for myself and for his caregivers*.”–LG-08-NOK (Close Friend) One-on-One Interview #2.

NOK believed the Last Gift team had worked diligently to ensure that the participants’ final wishes were met, such as the opportunity to contribute to impactful research.

“*I know [the study] meant leaving a legacy for sure that was something he talked about a lot and to kind of make sense of just his whole situation of having HIV and ALS at the same time*.”–LG-05-NOK (Partner) Focus Group #1.

“*When we became participants in the study I kind of stepped back to let [LG01] shine in the spotlight*. *I wanted it to be about him and I wanted him*, *you know*, *to know that I supported him in everything that he did*. *But I wanted it to be expressed through him and what he wanted and again his being a member of it and helping the community is… about the only thing with ALS that he could do is smile and this definitely made him smile*.”–LG-01-NOK (Spouse) Focus Group #1.

When asked about participant vulnerability, NOK reported that they did not feel Last Gift study participants were vulnerable, because participants were well-informed about what the study entailed. However, some participants felt that members of the public may stigmatize HIV research, thereby potentially rendering Last Gift study participants as vulnerable.

“*I can see how people might look at [the study] that way*, *but not at all because the option that everyone has throughout to say yes or no to anything*.”–LG-05-NOK (Partner) Focus Group #1.

“*I think that [study participants] were well advised as [to] what was happening and what was expected and I think that they went into [the study]*, *or [LG01] went into it*, *with full knowledge of what he was doing and did not feel that he was being taken advantage of*.”–LG-01-NOK (Spouse) Focus Group #1.

*“[S]ome people… would say … ‘I don’t know what the difference is wasting our money doing [research with] somebody that has [HIV]*,*’ you know so there was a little bit of they are vulnerable on that*. *But*, *you know*, *but I haven’t heard anything negative from anybody*.*”*–LG-04-NOK (Close Friend) One-on-One Interview #1.

Overall, NOK perceived the Last Gift to be an ethical study. Terminally ill participants were not perceived to be vulnerable because they had autonomy and were well-informed when providing informed consent. Notably, NOK reported that the Last Gift team helped ensure that participants could have their final wishes actualized. However, a NOK also noted that participants may potentially face vulnerability due to the stigma associated with HIV. NOK did not report ethical conduct issues associated with participation in the Last Gift study.

### NOK’s perceived benefits of the last gift study

NOK reported feeling supported by the Last Gift team when navigating the dying and grieving process. They perceived experiencing personal growth in their NOK role, as they learned more about HIV cure-related research and reflected on ways they, too, could contribute to their communities. NOK stated that, without support from the Last Gift team, they would have otherwise had no support in navigating the dying process of their loved one.

“*I feel that we have benefited even more than what [LG-08’s] contribution had to be*, *because the dying experience is just so overwhelming*.”–LG-08-NOK (Close Friend) One-on-One Interview #2.

“*The tremendous outpouring of love that I felt from everybody that was involved in the Last Gift project really helped me address [the grieving process] and deal with it and get through it and I am very happy that they were there for me*.”–LG-01-NOK (Spouse) Focus Group #1.

One NOK described the study team as a “24-hour hotline” because NOK could have immediate support any time they needed. The EOL process became more manageable for one NOK because they were confident that they had the necessary support. They felt comforted knowing they were not alone.

“*We would have just been out there by ourselves trying to figure things out on our own*. *But the Last Gift gave us like a 24-hour hotline that we could use and they actually became like a social group of friends that*, *you know*, *[LG08] got to know very well and a couple months before his death*, *so they were taking the walk with us as we sort of went into this vacuum of this unknown thing*.”–LG-08-NOK (Close Friend) One-on-One Interview #2.

For some NOK, this was the first time they had experienced the EOL process with a loved one. Having a support system such as the Last Gift Study team with experience in the dying process proved to be valuable.

“*[The Last Gift is a] supporting umbrella for somebody that didn’t know exactly what to expect*, *didn’t have very much experience with death and dying*, *and was one of the very few people that [LG08] asked to support him*. *So*, *for me personally… it was a big cushion there that I don’t know what I would’ve done if I didn’t have it*.”–LG-08-NOK (Close Friend) One-on-One Interview #2.

NOK also felt supported throughout the grieving process. Many noted the importance of receiving calls from Last Gift staff members who checked on their well-being during and after the Last Gift participant’s death.

“*It was nice to know that you guys were still thinking of us and it wasn’t like ‘okay it’s done now*, *next person*,*’ it was very*, *very nice to have contact still with you guys*.”–LG-05-NOK (Partner) Focus Group #1.

NOK reported positive experiences after confronting the death of a loved one—an experience they may have previously been uncomfortable with. Some NOK stated that they were not as frightened by death after supporting a loved one at the EOL and having the necessary support they needed throughout their role as NOK.

“*Prior to this experience I was very nervous about death*. *Now that I have gone through it*, *I feel much more comfortable about death and dying and even my own death*. *I sort of feel*, *like I told [LG08]*, *I wasn’t the one dying but I felt like I was in the back of the car with him*. *And so even though I wasn’t the person experiencing death myself*, *I feel the entire experience taught me what death was like*, *could be like*.”–LG-08-NOK (Close Friend) One-on-One Interview #2.

The study was a learning experience for NOK regarding the science and advancements behind HIV cure-related research.

“*[The Last Gift] has changed my life*, *it’s opened my eyes to what is being done in the scientific community and the HIV community*.”–LG-01-NOK (Spouse) Focus Group #1.

“*For me [the Last Gift] shows that we’re all in this together and that there’s people like still working really hard still to find the cure for HIV ‘cause we don’t really see that anymore I would say in the news it’s more just like donate to this cause with money or time but this is like we’re seeing it first hand*, *like very closely*, *the research being done*.”–LG-05-NOK (Partner) Focus Group #1.

Other NOK, after supporting a loved one through the Last Gift Study, were inspired to give back to their communities by sharing lessons learned. One NOK encouraged others to support someone through a difficult time.

“*I felt so proud of myself*. *I felt everyone should be*, *sort of*, *put in this situation where they have to help somebody else*, *so we organized a school-wide care fair instead of the regular science fair*. *So*, *every child had to do a service-learning project to better the world or someone or help someone in some way… Some of the children’s’ projects raised thousands of dollars*.”–LG-08-NOK (Close Friend) One-on-One Interview #2.

NOK reported several perceived benefits as a result of supporting a loved one through the EOL and participating in the Last Gift study. NOK felt they learned more about work conducted in the scientific community to combat HIV. After their participation in the Last Gift study, NOK also felt inspired to share lessons learned and contribute to a greater cause in their communities.

### Perceived drawbacks for NOK

When asked about drawbacks associated with the NOK role and NOK involvement in EOL HIV cure-related research, NOK acknowledged several potential drawbacks. These drawbacks included increased sadness and emotional stress related to the EOL process, conflicted wishes between NOK and the Last Gift participant, and a potential for invasiveness at the EOL.

Being confronted with the EOL process through research participation created sadness for one NOK because participation served as a reminder that their loved one was close to death.

“*It’s a lot of sadness*… *and there are again*, *like I say*, *I am proud of [LG07] for initiating this*.”–LG-07-NOK I (Grandmother) Focus Group #2.

*“[W]e were on a clock [for the body donation]*, *so it was*, *it was hard to be there and watch but being with family and friends that were there really helped to not lose it*.”–LG-05-NOK (Partner) Focus Group #1.

One NOK noted that intervening and suggesting that the Last Gift participant postpone blood draws at the EOL resulted in arguments between the NOK and the participant.

“*So*, *my feelings are I didn’t like that*, *me intervening to him*, *saying*, *‘You don’t have to [get your blood drawn]*.*’ And him saying*, *‘Yes I do*.*’ So we argued*. *Not*, *not a big argument*, *but [we] argued*. *And I backed off and I knew that he was adamant about it*.”–LG-04-NOK (Close Friend) One-on-One Interview #1.

NOK described sometimes feeling overwhelmed in their role as NOK. For one NOK, participating in EOL research while being the primary caretaker of a loved one nearing death felt like an additional responsibility.

“*I’m donating all my time and trying to take care of this guy and then I have to accommodate you guys as well*? *I felt a little bit*, *you guys should have communicated with me a little bit*.”–LG-04-NOK (Close Friend) One-on-One Interview #1.

Elderly NOK felt distressed by their physical inability to visit their Last Gift participant in hospice.

“*The fact that [LG07]’s up in hospice and he doesn’t really drive much anymore so we [grandfather and grandmother] have a difficult time getting up there to see him*. *I’d like to see him more*. *But*, *physically we haven’t been able to and I haven’t been able to in the last few months*.”–LG-07-NOK II (Grandfather) Focus Group #2.

Most participants reported the study did not encroach upon the EOL; however, one NOK reported the participant’s involvement with study procedures was stressful because they did not think the participant was in the right state to have their blood drawn.

“*I think he fell down*, *trying to get up and he just*, *he wasn’t able to walk very good at the time and really didn’t want you guys to come at the very end*. *It was the last thing*. *But didn’t have*, *didn’t have the whatever to say no*.”–LG-04-NOK (Close Friend) One-on-One Interview #1.

NOK reported that while perceived physical risks (to themselves or participants) were not associated with participating in the Last Gift study, the study may have brought emotional stress and psychosocial burdens involving terminally ill persons at the EOL. There was also a perceived potential for invasiveness if participants feel obligated to agree to study blood draws and interviews at the EOL.

### Inspiration from participants’ altruism

Throughout our focus groups, NOK expressed their support for the study and viewed it as an opportunity for participants to create further meaning at the EOL. The most common reason that NOK supported participation in the Last Gift study was that the study allowed participants to give their lives further meaning by helping others. One NOK reported feeling content knowing that participation in the Last Gift study allowed participants to serve a higher purpose.

“*It gave me the opportunity to see [LG01] further his life and know that he was happy doing something that previously there had been no opportunity for and so it made me real happy to know that he was able to further research with what he did*.”–LG-01-NOK (Spouse) Focus Group #1.

NOK also reported being proud of their loved one’s decision to enroll in the study.

“*Well I think we’re both [grandfather and grandmother] real proud of him because he had more than himself on mind when he*, *when he made this decision and we*, *we tried to help as much as we could and I*, *I admire him for it*.”–LG-07-NOK II (Grandfather) Focus Group #2.

In the NOK perspective, the Last Gift study was associated with a high level of altruism. NOK were proud and in admiration of their loved ones for committing their EOL to a cause with an aim to improve the lives of others and advance science.

### Suggested improvements for study conduct

NOK provided recommendations to make the study more focused on participants and their loved ones. One NOK suggested that to ensure the study maximally avoids invasiveness, thorough communication is necessary between NOK and study staff, especially as participants’ health status begins to decline.

“*Maybe talk to the person*, *like myself*, *so that the person that’s dying to see*, *you know*, *if they’re gonna be okay if we could come in*? *What do you feel*?”–LG-04-NOK (Close Friend) One-on-One Interview #1.

Another NOK admitted that the cremation process could be more seamless. They noted that the cremation process took longer than anticipated.

*“My only concern was the cremation seemed to take forever and I just wanted to get him home*.*”*–LG-01-NOK (Spouse) Focus Group #1.

Although several NOK did not have any suggestions to improve the Last Gift study, other NOK were able to identify areas that could enhance the study conduct. For better study implementation, NOK suggested that Last Gift study staff should prioritize improving their communication with NOK. NOK also perceived the cremation process to be more stressful than anticipated; therefore, they suggested that study staff help ensure the cremation process is as efficient as possible following rapid research autopsy.

## Discussion

This paper sheds light on the experiences of NOK involved in the Last Gift study at UCSD. Despite the expansion of EOL HIV cure-related research, there have only been a few studies investigating the experiences of NOK/loved ones in this setting [18]. Our initial paper on ethical considerations found that EOL HIV-cure research can be ethical when key issues are anticipated and participants’ lives are honored [5]. Our ethics paper also revealed the critical role of NOK in accepting their loved one’s EOL research participation [5]. In this paper, NOK demonstrated an accurate understanding of the clinical study and perceived the Last Gift study was conducted ethically and respectfully. Given the crucial role that NOK play in the EOL research process, their feelings and concerns, in addition to those of participants, must be considered. This qualitative study using focus group discussions (and guides) advances our understanding of the perspectives, experiences, and concerns NOK have when their loved ones elect to participate in HIV cure-related research at the EOL.

Through our discussions with NOK, we learned that NOK had an accurate and positive understanding of the Last Gift clinical study. Some mentioned that they themselves would participate in this study. While most NOK in our focus groups did not expect any clinical benefit or cure for their loved ones’ HIV or terminal illness, one NOK believed the Last Gift clinical team also served as the participant’s care team. Confusion on the distinction between clinical research and treatment, termed therapeutic misconception, may lead NOK to draw incorrect conclusions regarding their loved ones’ involvement in EOL research [19]. Therapeutic misconception is a widespread problem among research participants, often leading to the inability to obtain meaningful consent [19]. Avoiding misconception protects the integrity of the research process [20]. To combat the potential spread of misinformation, the Last Gift study team encouraged NOK to be actively involved with the study. Our study staff invited NOK to be part of the participants’ onboarding process to learn details directly from staff. Additionally, it is essential for study staff to clearly explain research study objectives to NOK, emphasizing that the research team will not be providing treatment or interfering with existing EOL care plans. While research staff do not provide palliative care, they may provide a sense of comfort to participants or NOK as a result of relationships that stemmed from the clinical study. By addressing therapeutic misconception with NOK, study staff can ensure a more ethically sound study.

NOK stated that their loved ones’ participation allowed research participants to have some control at the EOL because each participant was in charge of how they would ultimately serve their community: through their ‘last gift.’ According to Peter et al., vulnerability is defined as the state of being “incapable of protecting one’s own interests [21].” In general, NOK did not perceive Last Gift participants to be vulnerable because they considered participants to be well-informed of study implications, and participants were given the option to decline or withdraw participation in any part of the research process. Nevertheless, it was noted by NOK that HIV is still stigmatized which may contribute to vulnerability. In our cohort, we have found through separate interviews that stigma varies depending on the relationship participants have with their family [22]. HIV cure-related studies, like the Last Gift study, can also be perceived as opportunities to destigmatize HIV.

In our discussions, it was noted by a NOK that one participant did not feel comfortable declining blood draws, likely because he felt an obligation towards the study [23]. Among research participants, feelings of obligation may precipitate from a desire to reciprocate for care and attention received in the past [24]. Additionally, social desirability may compromise participants’ decisions, specifically when attempting to “look good to others,” thereby acting against their own interests [25]. It is imperative that research staff address any participants’ feelings of obligation or social desirability, explicitly stating that all aspects of the study are optional, and that there will be no repercussions for opting out.

While perceptions of benefits for participants in HIV cure-related research have previously been studied, this is the first time to our knowledge that a study is examining the perspectives of NOK specifically in small focus group settings [26]. Though the NOK of a loved one at the EOL oftentimes knows to expect an eventual death within their inner circle, they may not be prepared for the dying process [27,28]. In the Last Gift study, NOK described feeling overwhelmed by the dying process, especially because many had never cared for a loved one who was dying. NOK expressed gratitude for access to the Last Gift team, whom they could call at any hour for support. According to NOK, by calming their worries and guiding them through the EOL process, the Last Gift team gave them greater confidence and less stress. Existing literature has shown that EOL conversations between family members and physicians may contribute to reducing depression and alleviating grief during the bereavement process [23,29]. Additionally, in a study by Aoun and colleagues, family members who felt supported by palliative care staff before the death of a loved one had a more positive bereavement experience [30]. While our study did not provide palliative care, NOK expressed gratitude to Last Gift staff for remaining in contact with them and following up regularly. These efforts by Last Gift staff made NOK feel supported and valued. Thus, it is important for EOL research staff to prioritize communication with NOK, even after their formal participation in the EOL study has ended.

Research participation may be beneficial for bereaved NOK because it provides them a therapeutic opportunity to describe their experiences and voice their feelings [31]. Therefore, interviewing NOK about their experience in a research context may, in fact, serve to be beneficial in their grieving process. Similar to previous interviews with NOK, our participants also made note of the comfort they derived knowing that their loved ones’ participation gave them a sense of purpose and peace with their death [22]. Finally, in other HIV research studies, participants shed light on an unexpected benefit from their participation: increased knowledge about research [32]. NOK of Last Gift participants had a similar experience: participation as a NOK encouraged them to become more educated about living with HIV and HIV research. Serving as NOK for the Last Gift study inspired some NOK to reflect on ways they could contribute to their community and to future generations. These examples of personal growth on behalf of NOK emphasize the far-reaching and lasting impact of EOL HIV cure-related research.

Given the sensitive nature of EOL HIV cure-related research, it is important for research teams to also address the concerns and/or drawbacks of all stakeholders involved in the study. NOK participants revealed that being routinely confronted with the reminder of their loved ones’ approaching death was a source of sadness for them. Previous EOL studies have investigated the psychosocial effects of bereavement and viable mitigation strategies [33,34]. Studies have also shown that interventions such as distribution of bereavement pamphlets, professional support services, and educational palliative care interventions for families involved with the EOL can help attenuate the burden of bereavement and possibly decrease rates of depression [33–35]. Though these interventions were studied in the context of palliative care, it is still important for EOL research staff to be sensitive to NOK’s emotions and to be well-equipped to support them through the EOL process without interfering with participants’ clinical plans.

NOK in our study described potential conflicts that may arise between themselves and Last Gift participants. Previous research has shown that NOK may feel tension due to disagreements and miscommunication at the EOL [36–38]. In our study, NOK also noted feeling stressed about needing to accommodate study staff while caring for their loved one at the EOL. Having anticipated this possibility, the Last Gift research staff planned that informed consent for blood draws and rapid research autopsy would be “discussed with a next-of-kin/loved one in order to minimize concerns or conflicts during the research and at the time of death [5].” However, our discussions with NOK revealed that these interactions were not sufficient in preventing possible conflict. Thus, the Last Gift team needs to maintain diligent efforts to educate, accommodate, and involve NOK in all aspects of the study.

Oftentimes, NOK feel responsible for protecting their loved ones from pain and stress at the EOL [23,39]. In our study, while participants had provided informed consent to undergo frequent blood draws and interviews at the EOL, NOK still felt the urge to intervene when they sensed that frequent study procedures could pose a potential for discomfort, either physically due to blood draws, or emotionally due to inopportune timing of interviews. Overall, successful EOL studies require study staff to be extremely flexible and sensitive to both participants and their NOK.

Despite the potential perceived drawbacks, NOK viewed participation in an EOL HIV-cure related study as a way for participants to create further meaning in their lives by serving a higher purpose, namely participating in HIV research in hopes of helping find a cure for future generations. Altruism at the EOL has often been described similarly, as a last meaningful contribution to society [40,41]. In terms of motivation for research participation, altruism has been noted as an even greater motivator than self-gain for those at the EOL [40]. In our study, NOK expressed a sense of pride for their loved ones’ altruism. For some NOK, participants’ altruism also served as an inspiration which prompted NOK to think of ways they could also serve a higher purpose within their own means. These lessons emphasize the importance of prioritizing patient-centeredness in EOL HIV cure-related research, as research staff can ensure that participants’ altruistic wishes can be realized [42].

With regards to improving study conduct, several studies highlighted that effective communication between patients, NOK, and EOL clinical staff is critical at the EOL [43–45]. Likewise, in the research context, clear and consistent communication between NOK and study staff is key to effective and ethical implementation of EOL HIV cure-related studies. In our discussions with NOK, a perceived drawback of participating in EOL research was the potential for invasiveness. One NOK suggested that towards the participant’s EOL, it would have been helpful for study staff to consult with NOK to ensure appropriate timing of study visits. It is also known that preparing for death and funeral services are deeply important to both participants and their loved ones at the EOL [46]. Pentz et al, in their ethical considerations in EOL cancer research, reported that study staff must strive to minimize the delay of funerals and other negative effects of research with the recently dead [8]. While the Last Gift study covers cremation services for logistical purposes, the study team should still ensure the cremation process is conducted efficiently so loved ones can observe their loved ones’ death according to their plans and to reduce further distress. Additionally, our discussions with the AVRC Community Advisory Board suggested that a clear informed consent and information packet should be made available to NOK to avoid possible sources of conflict and misunderstanding. The Last Gift study staff recognize that cremation services engage with some religious traditions but not others, as such, the team will honor the wishes of the participant should they desire a traditional burial.

While our study adds to existing literature regarding psychosocial impacts of EOL research on NOK, it also emphasizes the need to better understand the challenges NOK face in the context of EOL HIV-cure related research. As such, in following ethical principles of beneficence and nonmaleficence, researchers must not only consider how to maximally reduce potential risks for NOK involvement in studies, but also how the study can serve as a positive experience [47]. A previous study demonstrated that bereavement significantly impacts the physical and mental health status of individuals who experience grief in the context of HIV-related death [48]. Another study showed that stress management interventions and social support groups not only serve to ameliorate the negative psychological impact of bereavement but also target “positive mental health and true wellness [49].” Considering previous studies on bereavement and our study results, which revealed that NOK may face increased sadness and stress when participating in EOL HIV cure-related research, we must ask the fundamental question: *what considerations are needed to ensure EOL research will not exacerbate the grief NOK already face*? Moving forward, we are encouraged to explore a broad range of methods to minimize the negative physical and psychological effects of EOL HIV cure-related research on NOK experiences.

We acknowledge several limitations associated with our study. First, given that six NOK responded to questions from our focus group guide, this study is limited by a small sample size at a single clinical research site. Thus, our results are not generalizable; however, the small sample size is consistent with research involving full body donations and rapid autopsies [6,8]. Given the small size of the study, we were also limited in the diversity of our sample. Most participating NOK were white and male, and as such, we were not able to study race, sex, or gender differences in perceptions; however, as more diverse NOK join, we seek to investigate these differences over time. In an effort to recruit a more diverse cohort, the Last Gift study team has recently made their website available in 10 languages. Participant and NOK brochures are now available in both English and Spanish as well. The very small sample size also limited our ability to analyze the data between NOK with Last Gift participants who were alive versus those whose participating loved ones had passed at the time of the small focus groups/interviews. Due to the small sample size, we did not achieve data saturation, and we will continue to monitor emerging themes in future research [50]. As our study grows, we will compare the perspectives and experiences of NOK at various stages of the study. Additionally, Last Gift participants had a choice of whether to refer a loved one for a small focus group. In turn, NOK had the option to decline participation in the small focus group portion of our study. Two Last Gift participants (LG02 and LG06) did not refer a NOK. Second, NOK who opted to participate in the focus group were generally more involved in the Last Gift study, giving rise to a potential for sampling bias. Therefore, results may be biased to reflect more positive or well-informed perspectives of NOK. Third, there is a potential for social desirability bias such that respondents refrained from sharing negative perspectives about the study. Fourth, our discussions posed a risk for emotional and death-related distress; therefore, our experienced focus group facilitators refrained from delving into deeply emotional topics associated with bereavement. Fifth, two research sessions had to be conducted as individual interviews due to logistical complications, but we remained flexible given the complex nature of EOL research and prioritized inclusion of as many NOK perspectives as possible; therefore, we decided to proceed with individual interviews using focus group guides.

Notwithstanding our study’s limitations, this research fostered discussions in a safe and flexible setting for NOK, thus yielding a rich source of data. Our findings may also inform other disease areas, such as EOL COVID-19-related research or EOL cancer research. We hope that our study can serve as a foundation to understanding the perspectives, experiences, and concerns of NOK in EOL HIV cure-related research and that additional literature will elaborate upon and enhance the work done in this paper.

**Table 4** summarizes key themes and findings from our discussions with NOK and possible implications for future EOL HIV cure-related research.

**Table 4:** Summary of findings and possible implications for future EOL HIV cure-related research studies involving NOK/loved ones (San Diego, California, 2019).

Summary of Findings	Possible Implications for EOL HIV Cure-Related Research
**Meaning of the Last Gift study** • There was an overall accurate understanding of the Last Gift clinical study, including the understanding that participants were not undergoing therapeutic or curative procedures. • NOK had a positive perception of the study and saw it as a compelling opportunity. This translated into the NOK’s own desire to participate in a study like the Last Gift.	• Teams involved in EOL HIV cure-related research should make every effort to involve NOK throughout the onboarding and study process to ensure that they are well-informed about all aspects of the research study. • To optimize ethical conduct, EOL HIV cure-related study teams must address therapeutic or curative misconception and ensure NOK understand that participation in the study will not confer any therapies or cures [19,20].
**Ethical conduct of the Last Gift study** • NOK expressed that the Last Gift was conducted ethically. • NOK did not perceive participants to be part of a vulnerable group given that they provided informed consent to participate in the study. NOK even noted that participating in the study was one of the few things participants had autonomy over during their EOL process. NOK believed the Last Gift allowed participants to leave their legacy and have their final wishes met.	• An ethical EOL HIV cure-related research study must ensure participants are well-informed about all aspects of the research study, even the option to refuse any part of the research process or study procedure without repercussion. • The research design must remain sensitive to feelings of obligation or social desirability that participants may experience. As such, it is imperative for EOL study staff to emphasize that all parts of the study are optional and there will be absolutely no consequences for opting out or withdrawing [23–25]
**Perceived benefits of Last Gift study for NOK** • Perceived benefits for NOK included feeling supported through the dying and grieving process, experiencing personal growth, learning about HIV and the dying process, and feeling inspired to support their communities.	• The psychosocial benefits of EOL HIV cure-related research for NOK must be appreciated. Given the team’s role in the EOL process and their experience with death and dying, study staff must ensure that NOK feel genuinely supported in their role. • To ensure NOK are and feel supported, study staff must prioritize communication with NOK both during and after their participation in EOL research process.
**Perceived drawbacks for NOK** • NOK perceived some concerns related to the Last Gift study such as minimizing sadness, emotional stress, conflict, and concerns around invasiveness. • Though NOK worried about potential for invasiveness, there were no perceived physical risk to themselves or study participants.	• EOL HIV cure-related research teams must remain keenly aware of the role they can play in minimizing the sadness and emotional stress associated with study participation. • By remaining alert to possible concerns, conflicts, and complications for NOK, EOL HIV research staff can constantly improve the study for both participants and their NOK. • Clear communication and thorough involvement of NOK in the EOL HIV cure-related research process plays a critical role in mitigating some of the drawbacks that NOK experience as a result of their study participation. Additionally, research teams must ensure that NOK’s thorough involvement in EOL research does not pose as an additional burden to NOK.
**Inspiration from participants’ altruism** • The Last Gift study was perceived to be a way for Last Gift participants to make their lives worth living because it allowed them to contribute to a greater cause. NOK felt proud and inspired that Last Gift participants had committed themselves to such an altruistic deed.	• By maintaining a participant-centered study, the research project must uphold the altruistic nature of EOL HIV cure-related research. It is also important for study staff to recognize the far-reaching impact of participants’ altruism and make every effort to help carry out Last Gift participants’ final wishes.
**Suggested improvements** • Some NOK suggested that research staff members make every effort to thoroughly communicate with NOK, especially as participants’ health status begins to decline. • Other NOK admitted that they felt the cremation process could become more efficient.	• It is imperative study staff are attuned to the needs of both participants and NOK. Research staff must engage in consistent and transparent communication with NOK through the course of the study. • For EOL studies that incorporate cremation services, it is critical study staff ensure that all necessary protocols and paperwork are in place prior to the rapid research autopsy and cremation [8]. • EOL HIV cure-related research studies must incorporate a clear informed consent process and information packet to prevent confusion and/or conflict for NOK.

## Conclusions

In conclusion, our qualitative focus group study highlights the perspectives, experiences, and concerns of NOK involved in the Last Gift study. Progress in EOL HIV cure-related research rests on community acceptance and involvement, including participants’ loved ones. To continue accounting for the voices and concerns of NOK in a participant-centered research study, we will conduct additional focus groups in the future and provide updates on findings in the literature.

## Supporting information

S1 Appendix Supplementary quotes. (PDF)

S2 Appendix COREQ checklist. (PDF)
