# Supplementary material for: Attitudes and perceptions of next-of-kin/loved ones toward end-of-life HIV cure-related research: A qualitative focus group study in Southern California
Source: PLoS One. 2021 May 7;16(5):e0250882. doi: 10.1371/journal.pone.0250882 (PMC8104928; doi:10.1371/journal.pone.0250882)
Supplement: S1 Appendix — (PDF) [file pone.0250882.s001.pdf]

S1 Appendix:

Themes	Quotes	NOK
**Meaning of the Last Gift Study**		
**Understanding of the Study by NOK/Loved Ones**	*For me it's still nice to make a contribution as well, now, because before I was just really on the sidelines and just caring for LG05 and so now it's kind of like a way to be close to him still*	LG-05-NOK
	Well, the research, uh, the answers it will probably give to someone down the line uh, by this study with us is going to mean a lot to me. And, uh, and I just feel like a lot of good will come out of it.	LG-07-NOK I
**Therapeutic Misconception**	When asked, *Okay, so your perception is that the Last Gift team was providing care to LG01?* NOK responded, *Yes.*	LG-01-NOK
**Positive Attitudes Toward the Last Gift Study**	*I- I am just so pleased and surprised that LG07 had consented to do this when it was a few years back and I understand it and I am proud of him.*	LG-07-NOK I
	I think it's important for me to carry it out to, to whatever the end of this study is supposed to be. He was part of the study, it was his study, so, um, it means a lot.	LG-04-NOK
	I saw a lot of positives though LG01 when he first found out about it he was very emotional that he had the opportunity to participate and he did his best every day to answer questions and donate blood when they needed it and he looked forward to the research that would be done on his body once he had passed on so he was very excited about it.	LG-01-NOK
**Ethical Conduct of the Last Gift Study**		
**Minimal Ethical Issues Perceived**	*No, I do not [perceive any ethical issues].*	LG-08-NOK
	I don't personally see any ethical issues or problems with getting close to [participants]. Like	LG-01-NOK

	I said for LG01 just be confirmed that he was loved by so many people was so special to him and I think that what you did, what you all did, was special to him and that he appreciated it. I don't think that he regretted your presence there with him at all.	
**Upholding of Participants' Wishes**	*[A]nd [the Last Gift study] was just- one of his things was that he wanted to educate people on what was happening with him, so yeah he was very open to everything*	LG-05-NOK
	He wanted to be cremated, but have his ashes with his dog. But that was a weird request, but you sort of figured out whether or not that would be possible.	LG-08-NOK
**Participant Autonomy Protects Them from Vulnerability**	*I mean, they are sick for sure. But you know, LG08 was very happy to participate in this study.*	LG-08-NOK
	It's something that he wanted to do and expressed his wishes that that's what he wanted done so I don't feel that he's in a vulnerable group at all.	LG-07-NOK I
**Perceived Benefits of the Last Gift Study for NOK**		
**Support for NOK in the Dying Process**	*The [Last Gift study] doctors came first thing in the morning. It was, like, 9 o'clock in the morning they were there… they gave him their personal telephone number."*	LG-08-NOK
	You've done more than your share and I know a lot of things you did. [S.C.G.,] you're a beautiful person and you make it really easy. I think they have the right people, the right person for the job here.	LG-04-NOK
**Support for NOK in the Grieving Process**	*[Y]ou know and even still to now, still feeling like I have a friend as opposed to just a stranger, you know. I welcome to see Susanna again today. So yeah, I still feel like I have the support network if I need it.*	LG-08-NOK

**Positive Experience with Confronting the Death of a Loved One**	*[I]t was- the death process the death was beautiful, I mean, if a death could be beautiful, that death was beautiful. It was very peaceful. And we all took a part in it.*	LG-04-NOK
**Learning More About HIV Research**	*I would like it for myself so, uh, I learned from that and you could get another customer.*	LG-04-NOK
**Inspiration for NOK to Also Give Back**	*So, you know I feel that LG08's life, uh, permeated into so many different ways to help so many different people that he knew there was going to be a care fair, um, but if he was able to see at the end what his life had resulted, what had resulted, he would have been shocked. I mean, one family alone raised $24,000 in toys for kids in sick hospitals.*	LG-08-NOK
	I think I talked to my daughter who lives here in San Diego, I would tell her, I think this is something I would do for myself if the study is still going. Definitely gonna donate- I definitely am a donor thing for the [HIV Neurobehavioral Research Center], but I think it's a good thing.	LG-04-NOK
**Perceived Drawbacks for Last Gift Study for NOK**		
**Increased Sadness**	*It's just a difficult situation talking about LG07 and his passing.*	LG-07-NOK I
**Conflict Between NOK and Participant**	*I said, 'Well, let me just call [the Last Gift team].' And he yelled at me and I said no. But I knew he didn't want to [have his blood drawn]. I regret that a little bit because it really, you know, he was - I think he died, uh, two or three days after that.*	LG-04-NOK
**Overwhelming Responsibility of NOK Role**	*I was, I was actually almost to the edge of my, my tolerance level. I had no more, I had nothing left. It was too hard. It was too hard for one person to take care of some- especially when they're in pain.*	LG-04-NOK

	I was going to the hospital about a year before when he had his ostomy surgery and it was so disruptive and unexpected and I got to a point where I just felt, this is so overwhelming. This is taking over my life and this whole thing is taking over my life.	LG-08-NOK
**Challenges for Elderly NOK**	*I'm just kind of mad because you can't just jump in a car and "hey let's go down and see LG07."*	LG-07-NOK II
**Concern for Invasiveness**	*I thought it was a little too pushy at the end, um, to get that last couple of interviews in there.*	LG-04-NOK
**Inspiration from Participants' Altruism**		
**Perceived Participants Serve a Higher Purpose**	*They're not just ending their life meaninglessly but they're able to do something that will hopefully provide a cure for HIV and I think that it's a wonderful opportunity*	LG-01-NOK
**Sense of Pride for Loved Ones' Altruism**	*Well, I think that the, the research that is being done on LG07 is going to help a lot of people that are going through the same thing –that it's gonna help them in the future uh, that uh, I'm just real proud of him for doing this and being in this study.*	LG-07-NOK I
**Suggested Improvements**		
**Active and Thorough Communication**	*[M]aybe he didn't relay it to me in the right way and so, um, maybe you should consult all people involved a little bit better, especially towards the end.*	LG-04-NOK
